# Determination of crystallographic intensities from sparse data

**DOI:** 10.1107/S2052252514022313

**Published:** 2015-01-01

**Authors:** Kartik Ayyer, Hugh T. Philipp, Mark W. Tate, Jennifer L. Wierman, Veit Elser, Sol M. Gruner

**Affiliations:** a Laboratory of Atomic and Solid State Physics, Cornell University, Ithaca, NY 14853, USA; b Field of Biophysics, Cornell University, Ithaca, NY 14853, USA; c Cornell High Energy Synchrotron Source (CHESS), Cornell University, Ithaca, NY 14853, USA; d Kavli Institute at Cornell for Nanoscale Science, Cornell University, Ithaca, NY 14853, USA

**Keywords:** X-ray serial microcrystallography, sparse data, reconstruction of diffraction intensity, *EMC* algorithm

## Abstract

X-ray serial microcrystallography involves the collection and merging of frames of diffraction data from randomly oriented protein microcrystals. The number of diffracted X-rays in each frame is limited by radiation damage, and this number decreases with crystal size. The data in the frame are said to be sparse if too few X-rays are collected to determine the orientation of the microcrystal. It is commonly assumed that sparse crystal diffraction frames cannot be merged, thereby setting a lower limit to the size of microcrystals that may be merged with a given source fluence. The *EMC* algorithm [Loh & Elser (2009 ▶), *Phys. Rev. E*, **80**, 026705] has previously been applied to reconstruct structures from sparse noncrystalline data of objects with unknown orientations [Philipp *et al.* (2012 ▶), *Opt. Express*, **20**, 13129–13137; Ayyer *et al.* (2014 ▶), *Opt. Express*, **22**, 2403–2413]. Here, it is shown that sparse data which cannot be oriented on a per-frame basis can be used effectively as crystallographic data. As a proof-of-principle, reconstruction of the three-dimensional diffraction intensity using sparse data frames from a 1.35 kDa molecule crystal is demonstrated. The results suggest that serial microcrystallography is, in principle, not limited by the fluence of the X-ray source, and collection of complete data sets should be feasible at, for instance, storage-ring X-ray sources.

## Abstract 2

A demonstration is given of three-dimensional crystal intensity reconstruction from sparse data, of a nature likely to be encountered in serial microcrystallography experiments at synchrotron sources.

## 1. Introduction   

Serial microcrystallography was developed as a way of taking advantage of the high fluence provided by X-ray free electron lasers to image small microcrystals (*ca* 1 µm^3^ or smaller) (Chapman *et al.*, 2011 ▶; Boutet *et al.*, 2012 ▶). Due to the short time duration of the pulse (<50 fs), the principle of ‘diffraction before destruction’ is applicable, where the pulse outruns most of the radiation damage. This allows the capture of relatively damage-free snapshot diffraction patterns. A large number of these patterns are captured by flowing a stream of crystals past the beam. Enough photons are scattered in this interval to allow indexing algorithms (White *et al.*, 2012 ▶) to determine the orientation of individual frames and to generate the three-dimensional intensity distribution of the diffraction.

This approach was reproduced at a synchrotron source (Stellato *et al.*, 2014 ▶) with larger crystals (135 µm^3^) and the same indexing method. However, with micron-sized crystals, around 100 times fewer photons would be scattered. Another possible application would be in the imaging of two-dimensional membrane protein crystals, given their weak diffraction (Feld & Frank, 2014 ▶). In both cases, there might be too few photons in a single frame to allow indexing of Bragg spots. The data may have the sparse nature of Philipp *et al.* (2012 ▶) and Ayyer *et al.* (2014 ▶), where it is impossible to recover the orientation of a single frame by itself. Fortunately, as in those cases, we show that one can apply the *EMC* algorithm to crystal diffraction (Loh & Elser, 2009 ▶) to simultaneously assign orientations probabilistically and solve for the three-dimensional intensities. Other algorithmic methods have been used (Fung *et al.*, 2008 ▶; Shneerson *et al.*, 2008 ▶; Kassemeyer *et al.*, 2013 ▶; Xu *et al.*, 2014 ▶) to assign orientations and extract the three-dimensional structure from two-dimensional snapshot(s), but they have been applied to much higher signal images.

To simulate the sparse data frame conditions from a 1 µm^3^ crystal at a storage-ring synchrotron source, we have performed an experiment with a large 400 µm crystal using a standard laboratory X-ray source with diffraction images recorded at a high frame rate. Each frame was acquired with the crystal in an arbitrary orientation around a single rotation axis. Even with an average signal level as low as 48 photons per frame (4.8 × 10^−4^ photons per pixel), we demonstrate successful recovery of orientation about the axis of rotation and reconstruction of three-dimensional intensities. We compare our reconstruction with a high-fluence data set where the orientations were recorded. We also examine the effect of background scatter on the quality of the reconstruction and the ability to recover orientations. The ability of the *EMC* algorithm to recover the three-dimensional intensities from sparse data, albeit from a large crystal, is an important step towards the development of *EMC*-based serial protein crystallography.

## 2. Reconstruction algorithm   

A slightly modified version of the *EMC* algorithm (Loh & Elser, 2009 ▶) was used to iteratively assign orientations and reconstruct the three-dimensional intensity distribution. One feature of this technique is that all regions of reciprocal space are treated equally. No particular preference is given to reciprocal lattice points. This is in contrast with the approach taken by indexing algorithms which emphasize the Bragg spots to the extent of ignoring the diffuse scattering. In the case of sparse data, most Bragg spots will produce no photons and some of the photons could be from non-Bragg background, making this approach impractical. A short review of the algorithm is given below.

The *EMC* algorithm has three steps in each iteration (expand, maximize and compress). Firstly, the space of available orientations is discretized to a finite number of angles. The expand and compress steps convert to and from the three-dimensional intensity distribution and the expected intensity at the detector in each of these orientations, which we call ‘views’. This is done using linear interpolation and the fact that detector pixels lie along the Ewald sphere. The maximize step uses the data frames to update the views using expectation-maximization, as described below.

Once the views have been obtained for each discrete orientation, the probability of a frame being generated by a view is calculated by assuming Poisson statistics for the number of photons recorded given an intensity. Thus, if the intensity at pixel *t* in view *r* is *W*_*rt*_, the probability *P* of frame *d* with *K*_*dt*_ photons at pixel *t* being generated by view *r* is given by 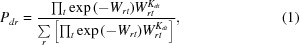
where the *K*_*dt*_! term has been omitted as it cancels out between the numerator and the denominator. Using these probabilities, the likelihood of maximizing an updated view *W*′_*rt*_ is given by 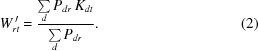
This intuitive update rule ends up being just the weighted mean over the data frames, with the weights being the probabilities calculated using the current model. These updated views maximize the likelihood of generating the data, given the probabilities *P*_*dr*_ calculated from the current model. The expand–compress cycle is necessary to impose consistency among different views by requiring that they come from a common three-dimensional model.

### 2.1. Three-dimensional *hkl* space   

One modification to the traditional algorithm is in the choice of space for the three-dimensional intensity distribution. The standard choice is Fourier space, where the slice representing the detector plane is the surface of a sphere passing through the origin (the Ewald sphere). Here, for reasons explained below, the best choice is *hkl* space, where the three axes represent the fractional coordinates with respect to the reciprocal unit cell. Thus, the reciprocal lattice points lie on a cubic grid with integer spacing, regardless of the unit-cell parameters. Unless the crystal has cubic symmetry, the detector pixels will no longer lie on the surface of a sphere but along some other surface. The pixel coordinates in this space can be calculated by using the basis vectors to determine the scaling and rotational transformation to the Ewald sphere surface.

With our choice of *hkl* space, the Bragg peaks all map to a cubic grid. Thus, while interpolating in the expand and compress steps, symmetry-related Bragg peaks see the same environment, which would not necessarily be the case if the basis vectors did not lie along the grid axes. This is important, because the maximize step is sensitive to slight variations among these peaks caused by interpolation errors. These errors are not a major factor in the case of non-Bragg intensity distributions where the variation is smooth on a one-pixel scale.

To define such a space, one needs to know the unit-cell vectors. These can be determined from the (high-signal) angle-averaged data, if not already known.

### 2.2. Initial guess   

As with any iterative algorithm, the initial-guess model must be specified. In this case, once the mapping of pixels to *hkl* space has been defined, the model is constructed by placing a three-dimensional Gaussian of random height at each lattice point. This cubic grid of random intensities is used as the input for the first iteration. Other than this initial guess and the use of *hkl* space, no other crystallographic symmetry is assumed during the reconstruction.

### 2.3. Rotation group subset   

In general, the set of views, *r*, is generated by sampling the three-dimensional rotation group uniformly. This is done with the help of unit quaternions. However, in cases where the crystal is rotated about a single axis and the relative orientation of the axis with respect to the crystal basis vectors is known, one can sample angles about just that axis. This was done in this experiment, where the axis was determined from the high-fluence data set using the *XDS* software (Kabsch, 2010 ▶).

## 3. Data collection   

The sample studied was a 400 µm sized small-molecule single crystal with chemical formula C_78_H_120_Mo_2_N_6_O (mol­ecular weight 1.35 kDa). It was mounted on the end of a glass fiber attached to a goniometer head, which allowed the crystal to be centered on the rotation axis. A rotation stage (Newport URS100) was set to rotate continuously at 0.1° s^−1^ during data collection. Although the angle of rotation was known for each frame, it was not passed to the reconstruction algorithm. The crystal was illuminated by a Mo *K*α beam generated by a Rigaku RU-H3R rotating anode set to 30 kV and 40 mA. Filtering was done using 200 µm of Zr foil to increase the fraction of *K*α radiation. The X-rays were focused to a spot of size 0.5 × 0.5 mm using Ni-coated Franks mirrors 1 m from the sample, with a beam convergence of 1 mrad and an intensity of 10^6^ photons s^−1^. The data were recorded using an MMPAD detector (Tate *et al.*, 2013 ▶) at a distance of 37 mm from the sample. Two data sets were collected, one with low fluence at 10 ms per frame and the other with high fluence at 500 ms per frame. The low-fluence data set was taken in groups of 1000 consecutive frames, with a time delay between sets to allow the frames to be written to disk.

**Figure 1:**
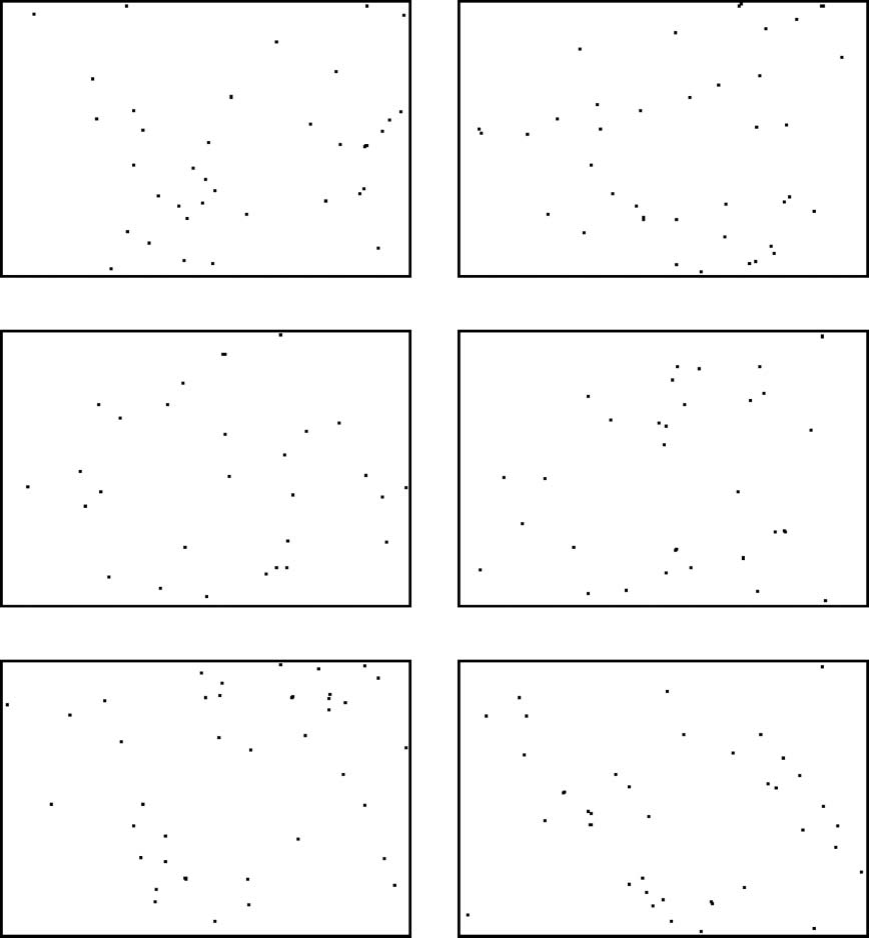
Six successive data frames obtained by collapsing 15 successive low-fluence frames together. Each collapsed frame has 48 photons on average. The locations of the photons have been emphasized to improve visibility. Although the crystal rotates slightly between these frames (0.015°), this rotation is below the instrumental divergence in the apparatus. At high fluence per frame, each of these frames would look similar. The observed difference between these frames, and the lack of any discernible Bragg pattern, show the data to be well within the sparse-data regime.

The data were then thresholded and photon counts were obtained using a procedure similar to that employed by Ayyer *et al.* (2014 ▶). In the low-fluence data set there were 4.3 million frames with an average of 3.2 photons per frame. Since the crystal rotated only 10^−3^° between two successive frames, multiple data sets were prepared by combining successive frames within a batch. Table 1 ▶ lists the details of the different data sets. Fig. 1 ▶ shows the first six frames from the 48 photons per frame data set.

**Figure 2:**
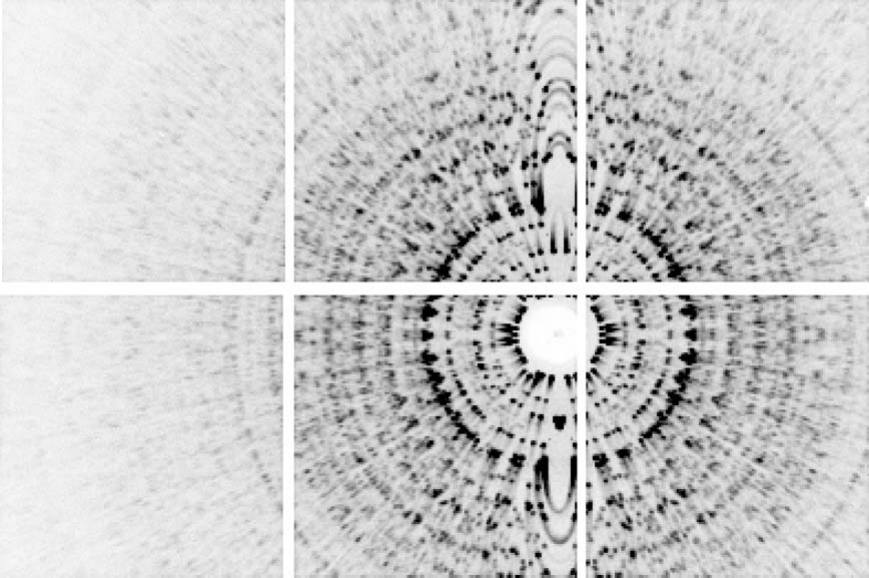
Angle-averaged pattern produced by summing over all frames in the low-fluence data set. The direct beam goes through the center of the beamstop and the rotation axis is vertical. Note the radial streaks caused by polychromaticity of the beam due to *Bremsstrahlung*. These streaks form arcs near the vertical rotation axis due to the curvature of the Ewald sphere. The white gaps in the image are the spaces between the six detector tiles in the 2 × 3 tiled array of the MMPAD detector.

Fig. 2 ▶ shows the angle-averaged pattern obtained by summing over all data frames. The radial streaks near the Bragg spots are caused by the polychromaticity of the beam. The arcs near the rotation axis are caused by the these streaks intersecting the curved Ewald sphere. Since the exact shape of the arc is very sensitive to the rotation axis, a region of the image within 11 pixels of the rotation axis was not used in the calculation of *P*_*dr*_.

## 4. Results   

**Figure 3:**
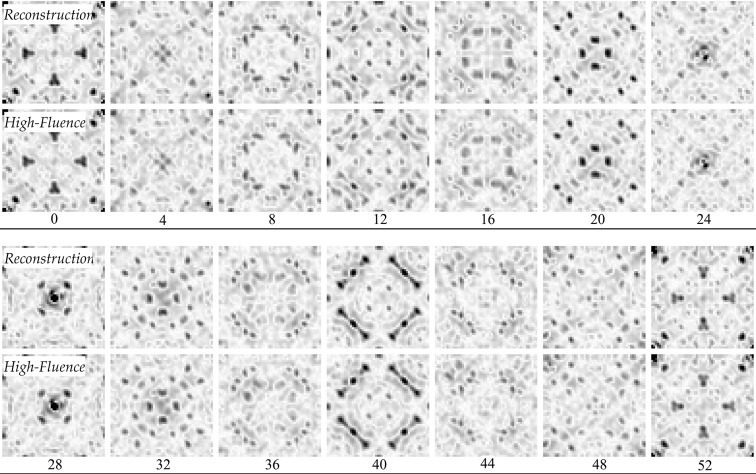
A comparison of slices through the three-dimensional Patterson maps generated from the high-fluence data set of known orientation and a low-fluence (48 photons per frame) reconstruction. The map was 53 × 53 × 53 voxels in size and every fourth slice is shown here, with the slice number shown below each pair.

**Figure 4:**
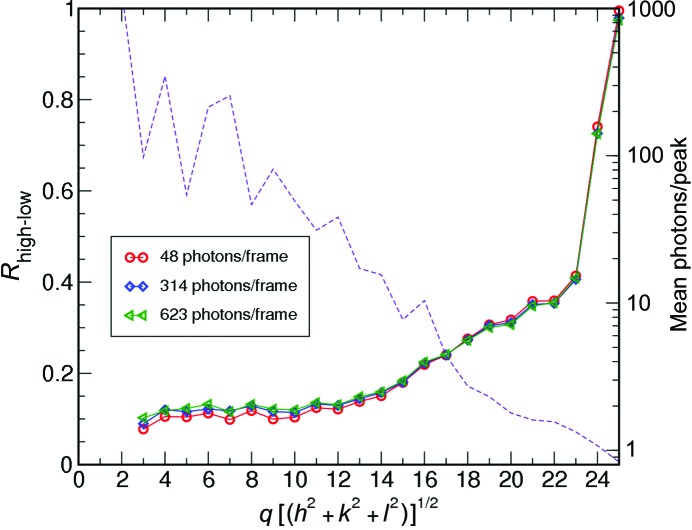
Plot showing *R*_high-low_ as a function of spatial frequency *q*. This quantity (defined in §4) is calculated by comparing a low-fluence reconstruction with the high-fluence assembly using the known orientations. The three different reconstructed data sets mentioned in Table 1 ▶ have been plotted. The dashed line represents the mean number of photons per Bragg peak as a function of *q* for the high-fluence data set.

The crystal analyzed had a body-centered cubic (b.c.c.) unit cell with a lattice constant of 21.47 Å in space group 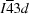. The unit-cell parameters were taken as a given and used to generate the mapping to *hkl* space (§2.1). The high-fluence data set with known orientations was used to generate a reference three-dimensional intensity model. This was compared with the reconstructions from different low-fluence data sets by comparing the Patterson maps, which were generated as follows. First, the intensities were integrated in a small sphere about every *hkl* point. The three-dimensional *hkl* grid of intensities was then inverse Fourier transformed to generate the electron-density auto-correlation function, which is the Patterson map. Fig. 3 ▶ shows a comparison of the maps for one particular data set (48 photons per frame). For a quantitative comparison, we have calculated an *R* factor between the Fourier amplitudes of the low- and high-fluence reconstructions (*R*_high-low_), shown in Fig. 4 ▶. This quantity was calculated as follows 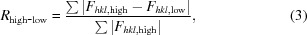
where *F*_*hkl*_ = (*I*_*hkl*_)^1/2^ and *I*_*hkl*_ refers to the integrated intensity at Bragg peak (*h*, *k*, *l*). We observe that, at low resolutions, this *R* factor is near 0.14. At high *q* [= (*h* ^2^ + *k* ^2^ + *l* ^2^)^1/2^], the lack of good statistics in the high-fluence data set leads to a large value. This is illustrated in Fig. 4 ▶ by the dashed line, which represents the mean number of photons per peak in a given resolution shell.

### 4.1. Dependence on photons per frame   

**Figure 5:**
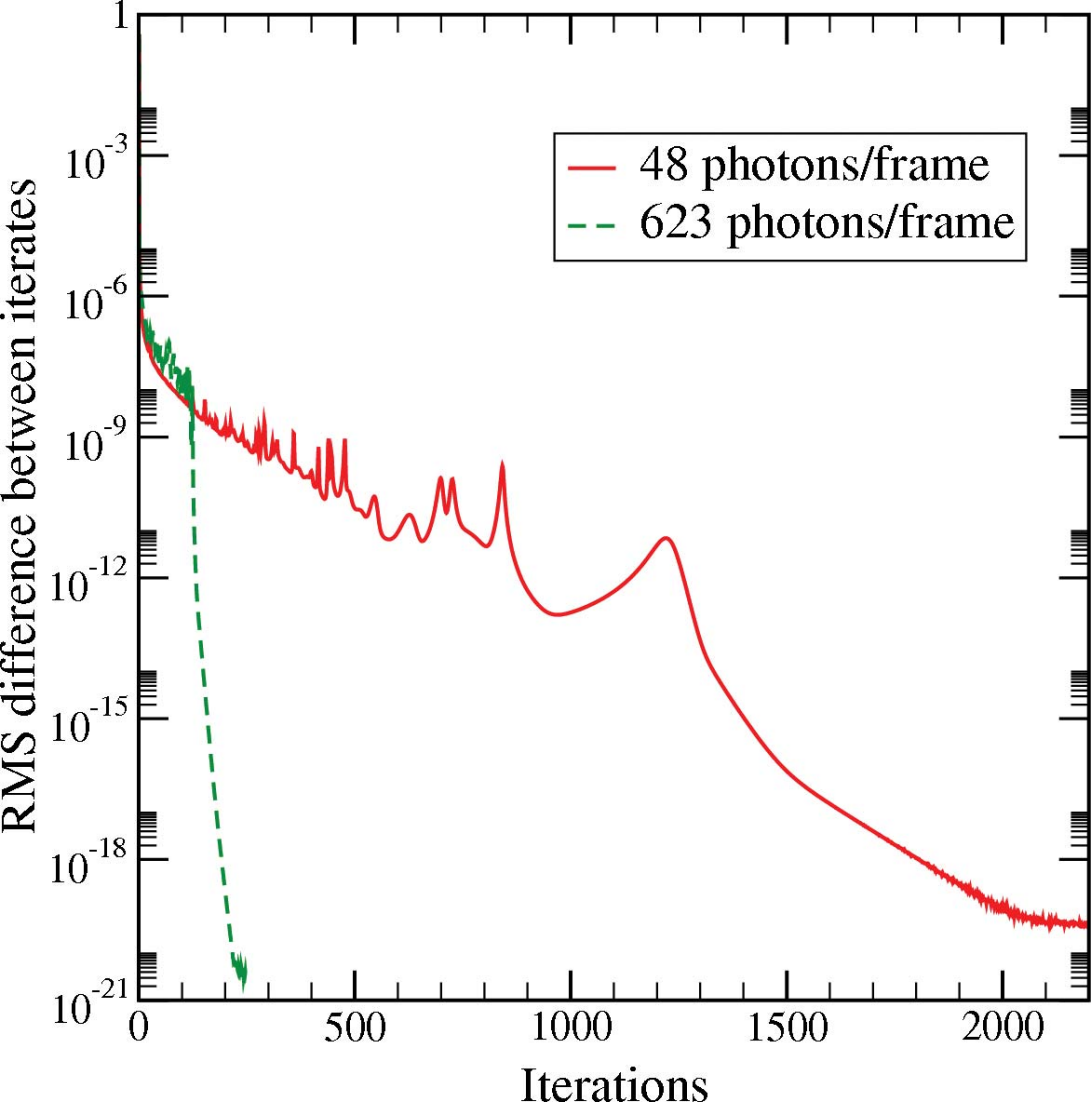
A plot showing the difference between successive iterates as a function of iteration number. The units on the logarithmic vertical are arbitrary; for reference, the lower limit corresponds to the floating-point precision of the computation. Two data sets are shown, with 15 collapsed frames (48 photons per frame) and 200 collapsed frames (623 photons per frame). The sparser data set takes much longer to converge and the slope of the curve in the last few iterations is strongly related to the number of signal photons per frame.

As mentioned in §3, the crystal is rotated by 10^−3^° over one frame. This allows us to collapse successive frames, as they come from almost identical orientations. Using this method, we could study the effect of the number of photons per frame on reconstruction quality while keeping other parameters the same. One effect of decreasing the number of photons per frame was that it took more iterations to reach convergence. For less than 48 photons per frame, the reconstruction did not converge at all. Above this threshold value, the reconstruction quality was independent of the number of photons per frame. However, it took many more iterations, as can be seen in Fig. 5 ▶ and Table 1 ▶. This is consistent with the observations in simulations with speckle intensity patterns (Loh & Elser, 2009 ▶). The threshold value itself is lower because of the different distribution of the intensity in this case (concentrated in Bragg peaks as opposed to large smooth speckles). While the situation might be affected by other factors, such as beam monochromaticity and crystal homogeneity, the rate of convergence will still be principally determined by the number of photons per frame.

### 4.2. Addition of background   

The large non-hydrated crystal that we used provided relatively little background scattering compared with the Bragg spots. To study the effect of uniform background on the quality of the reconstruction, additional photon counts were added, with a Poisson distribution of uniform mean, to each data frame of the 314 photons per frame data set. Except in the cases of extreme background, there is no effect on the orientation recovery. The weak highest-resolution peaks are lost as they are drowned out by the noise in the background. This is an unavoidable aspect of crystallography.

**Figure 6:**
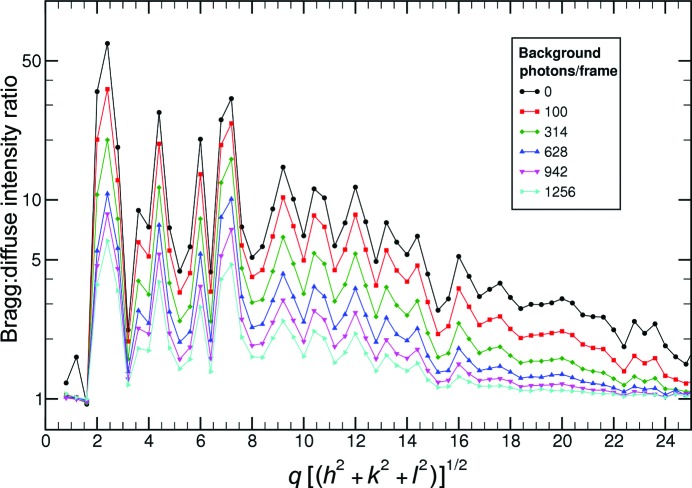
Plot of the Bragg to diffuse intensity ratio as a function of spatial frequency *q* for various amounts of added photons to simulate background. A high ratio indicates that the orientations have been correctly identified and most of the intensity is in Bragg peaks, whereas a ratio near 1 would mean that the peaks are not resolved over background. There were 314 photons per frame in the base data set. Even with 400% background, the low-resolution peaks were resolved, as seen in the 1256 photons per frame plot.

To demonstrate the successful recovery of orientation at different background levels, the ratio of average intensity per voxel in the neighborhood of a Bragg point to the average intensity in the diffuse region is plotted *versus* reciprocal length, *q*, in Fig. 6 ▶. If this ratio is close to 1, the Bragg peaks do not stand out over the background. As the plot shows, even with high background, the strong low-resolution peaks are successfully recovered. However, as expected, the weak high-resolution peaks are lost.

### 4.3. Computational details   

The reconstruction was performed on a single machine (Intel Xeon E5-2640 @ 2.00 GHz with 128GB RAM running Scientific Linux) and took around 15 s per iteration using 20 cores. The three-dimensional *hkl* grid was chosen to have a cubic lattice constant of 10 voxels. The detector geometry then meant that the grid was 366 × 366 × 366 voxels in size. The initial model was generated by placing a three-dimensional Gaussian of random height and width 2 voxels centered at each reciprocal lattice point satisfying the b.c.c. selection rule (*h* + *k* + *l* = even). A similar procedure was performed during peak integration, when the values in a sphere of radius 2 voxels around each peak were summed to calculate the Bragg intensity.

Since the rotation axis with respect to the crystal axes was taken to be a given, 1150 (≃ π × 366) equally spaced orientations were sampled about that axis.

We consistently observed that, if the algorithm converged, it produced very similar maps. Thus, convergence was taken to be an indicator of a successful reconstruction. Here, iterative convergence occurs when the iterate stops changing from one iteration to the next. Some convergence plots are shown in Fig. 5 ▶.

## 5. Conclusions   

We have shown that the three-dimensional diffraction intensity distribution can be calculated from a large number of sparse data frames, each with unknown orientation. This result bodes well for the possibility of performing serial crystallography with micron-sized or smaller microcrystals at synchrotron sources. Complications will no doubt arise when the method is applied to protein crystals of decreasing size and an additional degree of rotational freedom, as we intend to do in follow-up studies. Still, it is promising that successful reconstruction of the three-dimensional intensities has been shown for a signal level as low as 48 photons per frame.

We also observe that the addition of relatively high levels of uniform background (400%) does not affect orientation recovery. This is important, as some base level of background scattering is unavoidable with protein crystals due to the solvent internal to the crystal. As is the case in all protein crystallography experiments, the background reduces the resolution as higher order peaks are drowned out. Reducing the background requires minimizing the amount of material in the beam path. Fortunately, it is possible to reduce the background to insignificant levels by appropriate X-ray optics, vacuum paths and graphene windows surrounding the crystal stream (Wierman *et al.*, 2013 ▶). For example, one can envision flowing a filtered set of uniformly sized microcrystals down a minimally sized tube equipped with graphene X-ray windows in an otherwise totally vacuum environment. If the exposure times are longer, fast-framing detectors (Koerner & Gruner, 2011 ▶; Johnson *et al.*, 2014 ▶) can be used to restrict artificially the net degree of angular diffusion over an exposure.

One feature of the serial crystallography experiment not replicated here is the collection of data from all orientations in three dimensions. Reconstruction from the full rotation group was studied in simulations by Loh & Elser (2009 ▶) for aperiodic structures with speckle intensity distributions. There, it was shown that the number of photons per frame required for successful reconstruction grows only logarithmically with the number of orientational samples. Although the total number of photons required for a complete data set with a good signal-to-noise ratio and good resolution will be higher than what was collected here, with the fluence available at third-generation X-ray sources one should be able to collect a complete data set of similarly sparse nature with micron-sized crystals. This suggests that sub-micron room-temperature serial microcrystallography should be feasible. Experiments to examine this prediction will, no doubt, be performed.

## Figures and Tables

**Table 1:** Properties of various data sets generated by collapsing successive frames Before collapsing, the frames were in batches of 1000 contiguous frames with gaps. There were also some rejected frames which had a very high photon count caused by incorrect detector offsets. The number of iterations required for convergence depends upon the random start, so the numbers given here are approximate and are used to highlight the trend.

Collapsed frames	No. of collapsed frames	Photons per frame	Iterations to converge
1	4321197	3.22	
10	434420	32.00	
15	290541	47.85	2200
100	44221	314.41	400
200	22321	622.88	250
